# Multicondition and multimodal temporal profile inference during mouse embryonic development

**DOI:** 10.1101/gr.279997.124

**Published:** 2025-10

**Authors:** Ran Zhang, Chengxiang Qiu, Galina N. Filippova, Gang Li, Jay Shendure, Jean-Philippe Vert, Xinxian Deng, William Stafford Noble, Christine M. Disteche

**Affiliations:** 1 Department of Genome Sciences, University of Washington, Seattle, Washington 98195, USA;; 2 eScience Institute, University of Washington, Seattle, Washington 98195, USA;; 3 Department of Laboratory Medicine and Pathology, University of Washington, Seattle, Washington 98195, USA;; 4 Brotman Baty Institute for Precision Medicine, University of Washington, Seattle, Washington 98195, USA;; 5 Howard Hughes Medical Institute, Seattle, Washington 98195, USA;; 6 Allen Discovery Center for Cell Lineage Tracing, Seattle, Washington 98109, USA;; 7 Seattle Hub for Synthetic Biology, Seattle, Washington 98109, USA;; 8 Owkin, New York, New York 10010, USA;; 9 Paul G. Allen School of Computer Science and Engineering, University of Washington, Seattle, Washington 98195, USA;; 10 Department of Medicine, University of Washington, Seattle, Washington 98195, USA

## Abstract

The emergence of single-cell time-series data sets enables modeling of changes in various types of cellular profiles over time. However, because of the disruptive nature of single-cell measurements, it is impossible to capture the full temporal trajectory of a particular cell. Furthermore, single-cell profiles can be collected at mismatched time points across different conditions (e.g., sex, batch, disease) and data modalities (e.g., scRNA-seq, scATAC-seq), which makes modeling challenging. Here, we propose a joint modeling framework, Sunbear, for integrating multicondition and multimodal single-cell profiles across time. Sunbear can be used to impute single-cell temporal profile changes, align multi–data set and multimodal profiles across time, and extrapolate single-cell profiles in a missing modality. We apply Sunbear to reveal sex-biased transcription during mouse embryonic development and predict dynamic relationships between epigenetic priming and transcription for cells in which multimodal profiles are unavailable. Sunbear thus enables the projection of single-cell time-series snapshots to multimodal and multicondition views of cellular trajectories.

Rapidly improving single-cell sequencing technologies now allows us to characterize diverse and dynamic cellular processes. Multimodal time-series measurements of a population of cells can profile changes in gene expression, chromatin accessibility and structure, and DNA methylation as the cells carry out essential biological functions, differentiate, traverse the cell cycle, degenerate, or undergo stages of disease progression.

Although such measurements have proven to be immensely valuable, they suffer from a fundamental limitation: Because sequencing measurements are usually disruptive, it is impossible to trace a given cell's behavior across time. In addition, single-cell profile inference suffers from heterogeneity of biological samples (e.g., female vs. male, disease vs. control) and data types (e.g., scRNA-seq, scATAC-seq), as well as limited sample availability. Consequently, a method capable of accurately imputing unobserved single-cell measurements—essentially answering questions such as, “what would the gene expression of this particular cell be if we had measured it at a different time point, or in a different experimental condition, or using a different measurement modality?”—would be immensely valuable in understanding the temporal dynamics and regulation of the behaviors of individual cells across biological conditions.

Many analytical techniques have been developed to model temporal processes from single-cell data. For example, pseudo-time-based methods infer the ordering of cells along a one-dimensional or branching trajectory (Trapnell et al. 2014; Tanay and Regev 2017; Saelens et al. 2019). However, these methods do not perform imputation per se, and the relationship between the inferred pseudotime and actual time is left unspecified. Methods using RNA velocity to derive single-cell trajectories assume that nascent RNAs represent the future state of a cell (Lange et al. 2022; Li et al. 2023). However, RNA velocity can be noisy and cannot be applied to other data modalities, and these methods are focused on ranking cells instead of predicting cellular profiles in missing time points.

In this work, we focus on methods that are specifically designed to integrate and extrapolate single-cell time-series measurements (Table 1). For example, optimal transport-based methods, such as Waddington-OT (Schiebinger et al. 2019), TrajectoryNet (Tong et al. 2020), and TIGON (Sha et al. 2024), operate on pairs of measurements in a time-series, with the goal of aligning cells from two neighboring time points under the assumption that a single cell's profile changes minimally between time points. Alternatively, neural ordinary/stochastic differential equation (ODE/SDE)–based methods, such as PRESCIENT (Yeo et al. 2021), RNAForecaster (Erbe et al. 2023), MIOFlow (Huguet et al. 2022), FBSDE (Zhang et al. 2024b), and scNODE (Zhang et al. 2024a), assume that each cell develops autonomously and that the cell's future state is determined based on the cell's current expression profile. Autoencoder models either assume that time is an additive variable in the embedding space (Lotfollahi et al. 2023) or are coupled with optimal transport or ODE-based methods to optimize nonlinear cell projection and cross-time alignment (Tong et al. 2020; Huguet et al. 2022; Zhang et al. 2024a). Graph-based methods, such as GraphFP (Jiang et al. 2022), perform dynamic inference on top of cell graphs. However, most of these existing methods are focused on a single data modality (e.g., single-cell RNA-seq) or biological condition. Recently, Moscot.time was proposed to jointly perform optimal transport on multiomics data (Klein et al. 2025). However, optimal-transport-based methods are aimed at matching cells between time points but not temporal profile inference at missing time points. To our knowledge and as shown in Table 1, no existing method is designed to predict temporal profiles of single cells at missing time points across conditions or modalities.

**Table 1.** Methods for integrating and extrapolating single-cell time-series data

	OT	ODE	AE	OT/ODE+AE	Graph	Multimodal OT	Sunbear
Optimized in the original data space			✓	✓			✓
Learns from distal time points		✓	✓	✓	✓	✓	✓
Does not require cell-type labels	✓	✓		✓	✓	✓	✓
Corrects for batch effects							✓
Models multimodal data						✓	✓
Performs imputation on missing conditions		✓	✓				✓
Performs imputation on missing modalities							✓

(OT) Optimal transport, (ODE) ordinary differential equations, and (AE) autoencoder.

We thus set out to create a method that is capable of modeling various types of cellular profiles—gene expression, chromatin accessibility, DNA methylation, etc.—while capturing trends over time, between modalities, and across experimental conditions at single-cell resolution. We were particularly interested in the cross-modal setting in which, for example, one data modality is “undercharacterized”; that is, we are missing measurements of that modality in one or more conditions or time points of interest. Although several existing methods have been developed for this type of cross-modal inference, they were developed for bulk analysis and have not been evaluated in the single-cell setting. For example, Sagittarius performs cross-species and cross-cell-line inference of bulk transcriptomic profiles through a transformer model (Woicik et al. 2023), and *chronODE* integrates bulk multiomics time-series data to model the temporal dynamics of gene and chromatin features (Borsari et al. 2025).

Following the lead of several existing methods (Sohn et al. 2015; Lopez et al. 2018), we hypothesized that a type of deep neural network known as a conditional variational autoencoder (cVAE) should be capable of accurately modeling a time-series data set consisting of multiple single-cell data conditions and modalities. Here, we propose Sunbear, a cVAE that incorporates continuous-time embedding to integrate data sets from unmatched time points and infer temporal profiles at any intermediate time point.

## Results

### Sunbear model overview

**Figure 1.**
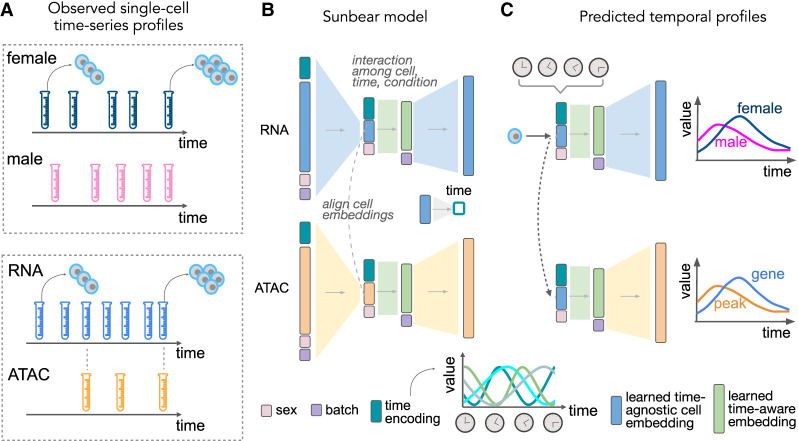
Sunbear framework. (*A*) Sunbear takes as input a collection of measurements of single cells at multiple time points in two or more biological conditions (*top*) or data modalities (*bottom*). (*B*) During the training phase, Sunbear learns to decompose the original time-series profiles into four components: cell embedding, time point, batch, and condition. Batch and condition factors are represented by one-hot encodings. The time factor is represented by a sinusoidal encoding. The cell embedding is learned from the original profile and is conditionally independent of the other factors. In the multimodal setting, cell identities are aligned between data modalities. (*C*) In the prediction phase, Sunbear concatenates the query cell's identity factor while varying other factors to impute the cell's profile across time and conditions. By sharing cell embeddings across modalities, Sunbear allows joint temporal modeling of multimodal profiles.

Our aim was to produce a model that would take as input a time-series of single-cell measurements from two or more different data modalities and predict, for any cell in the input, its multimodal profile as if the cell had been measured at some other time point (Fig. 1A). To accomplish this goal, we created Sunbear, which is a cVAE that includes a continuous-time embedding and incorporates features within the model to enable cross-modality data set matching (Fig. 1B). To provide additional flexibility, Sunbear also includes embeddings to represent experimental or biological conditions (e.g., sex, batch, or perturbations).

Intuitively, through the encoder framework, Sunbear learns cell embeddings (default dimension: 50; see hyperparameter tuning strategies in Methods subsection “Hyperparameter tuning”) that represent cell identities while ensuring that these embeddings are conditionally independent of the time, study conditions, and batch effects. To take advantage of the continuous nature of time and enable learning diverse temporal patterns, Sunbear incorporates a time factor using a sinusoidal encoding (default dimension: 50). A time-aware cell embedding is then learned by integrating cell identity and time information, which is further projected back to the original gene expression space through a decoder. Critically, Sunbear is capable of learning nonlinear relationships between time and cell embedding, because the model is optimized to reconstruct accurate cellular profiles for all cells across all time points (Methods subsection “Modeling scRNA-seq temporal profiles”).

In the multimodal setup, a second cVAE is built for scATAC-seq data. To ensure accurate cross-modality integration and inference, Sunbear forces cell embedding alignment between data modalities. Furthermore, to augment temporal inference in the data modality when sparser time points observed (i.e., scATAC-seq in our example), Sunbear forces the temporal interaction layer (Fig. 1B, light green box) to be shared between modalities, with the assumption that the interaction effect among cell, time, and condition should not vary based on the data type a cell is measured in.

Once the model is trained, we can continuously vary the time factor to infer smooth trajectories in cellular profiles across time. Furthermore, because Sunbear can be jointly optimized on multimodal profiles, we can couple encoders of, for example, gene expression (scRNA-seq) with decoders of chromatin accessibility (scATAC-seq) to enable the prediction of missing scATAC-seq profiles across time (Fig. 1C).

### Sunbear can predict a cell's transcriptional profile at missing time points and missing conditions

Because of the lack of ground-truth measurements of a cell's expression changes across time, we first carried out a simulation study to make sure Sunbear can capture different temporal trends at single-cell resolution. In this simulation study, we are interested in testing two aspects of the model.

First, given diverse temporal patterns of gene expression and noisy single-cell measurements, can Sunbear recapitulate single-cell gene expression changes across time? To achieve this goal, we simulated a data set with a relatively large amount of noise (standard deviation of three) and no pseudotime differences among cells (time delay: zero) (Supplemental Methods, “Simulation studies”). We then trained Sunbear on the resulting data set. For the prediction, we started with a single cell's profile from time 0 and varied the time encoding to predict the cell's corresponding profile at continuous time points. We then visualized randomly selected genes’ expression changes (Supplemental Fig. S1). Our result suggests that Sunbear is able to capture temporal patterns of gene expression across different cell types and genes.

Next, we are interested in testing whether Sunbear can recapitulate the heterogeneous nature of cell types at single-cell resolution. For each cell type and each time point, we assume that 100 cells have unsynchronized pseudotimes, in which each pair of neighboring cells differs by 0.002 days, and there is a total of 0.2 day differences in pseudotime. We set the standard deviation of noise to one to prevent the noise overriding the signal of cellular differences. In this case, we systematically compared the performance of Sunbear and baseline predictions on each held-out time point, 6.75, 7.25, 7.75, and 8.25, across all genes and cells (Supplemental Methods, “Simulation studies”). Overall, our simulation results suggest that Sunbear can accurately capture individual cell-level differences (Supplemental Fig. S2).

**Figure 2.**
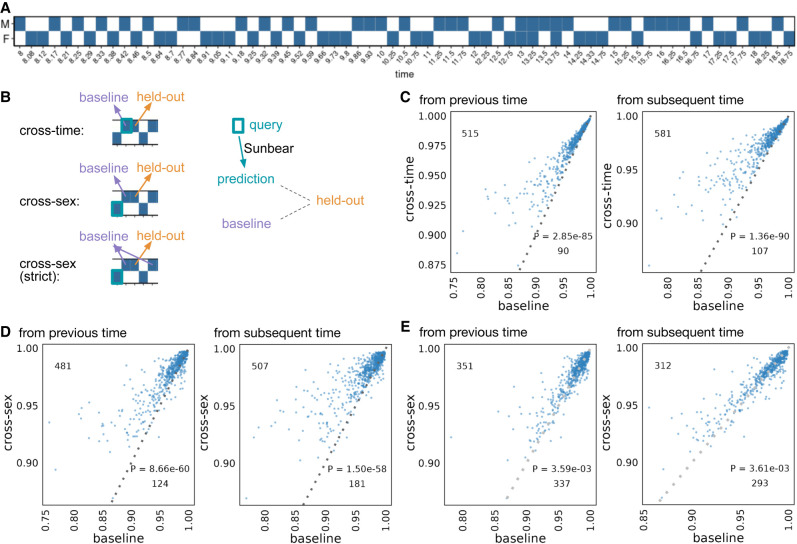
Single-cell profile inference across time and conditions. (*A*) Sunbear is trained on scRNA-seq profiles of whole-mouse embryos collected at alternating sexes along developmental time points (Qiu et al. 2024). (*B*) Sunbear is validated in three scenarios. In each scenario, one data block is held out from the training, and Sunbear is used to predict the profile of the missing block based on cells in the query block (outlined in turquoise). Sunbear's prediction is compared against the baselines using the held-out block's nearest existing measurements with the desired sex factors. The pseudobulk Pearson's correlation per major cell trajectory is calculated between the held-out profile and predictions/baselines. (*C*) Cross-time evaluation: query and baseline are selected either from the closest previous time point (*left*) or from the closest subsequent time point (*right*). The pseudobulk Pearson's correlation between the original held-out profile and predicted (*y*-axis) and baselines (*x*-axis) is plotted for each major cell trajectory in each held-out time point. Each dot represents a cell trajectory per held-out time point, and numbers indicate the number of dots *above* and *below* the diagonal line. *P*-values are calculated by a one-sided Wilcoxon rank-sum test. (*D*) Cross-sex prediction: similar to that in *C*, except query cells are selected from the opposite sex to the held-out data. (*E*) Cross-sex prediction: similar to that in *D*, except we enforce a strict baseline model by taking the mean of the previous and subsequent time point per cell trajectory.

We then set out to test Sunbear's ability to predict a cell's gene expression profile at missing time points. For this analysis, we used a set of 1 million cells randomly sampled from our recently published time-series mouse embryonic development data set, which consists of 12 million cells collected at 67 time points from embryonic day (E) 8 through E18.75 (Fig. 2A; Qiu et al. 2024). To validate Sunbear's performance at cross-time and cross-condition prediction, we held out one time point at a time and trained a model on the remaining time points (see the Methods subsection “scRNA-seq temporal inference evaluation”). In Sunbear, the time point's information is encoded in a latent “time” factor, which is concatenated with the cell identity factor to impute the scRNA-seq profile of each cell. The model's training procedure is designed to encourage the cell identity factors to be invariant of time. Therefore, by fixing the cell factors and varying the time factors, we can predict scRNA-seq profiles in a missing time point.

To quantitatively evaluate Sunbear's prediction performance, we would ideally like to measure Sunbear's ability to predict how a single cell's expression profile changes across time. However, because single cells are destroyed after sequencing, this type of evaluation is impossible. We therefore adopted an alternative approach that relies upon the cell-type annotations created by the authors of the original study. These annotations allow us to ask how well each cell-type-specific pseudobulk profile predicted in the missing time point agrees with the true cell-type-specific pseudobulk profile that is held out from training (see the Methods subsection “scRNA-seq temporal inference evaluation”).

Specifically, to validate Sunbear's performance on temporal inference, we asked whether the profile of each cell in the held-out time point can be recapitulated by using cells from the corresponding cell type at a neighboring time point. To do that, we compared Sunbear's prediction (i.e., by varying the time factor of the query cell type at a neighboring time point to the held-out time point) with the baseline prediction, which is the pseudobulk profile of cells in the time point immediately before or after the held-out time point. Even though Sunbear did not see the cell-type information during training, it was able to predict scRNA-seq profiles in an unseen time point better than the baseline methods in the majority of cell types and time points (Fig. 2B, “cross-time”; Fig. 2C).

In addition to modeling a continuous variable such as time, Sunbear is capable of modeling discrete factors corresponding to various types of experimental conditions. This functionality allows us to swap the condition factor to predict a cell's profile in a missing time point and in a different condition, thus making direct comparisons of gene expression profiles across sexes. We used this functionality to predict sex-specific single-cell profiles at missing time points (Fig. 2B, “cross-sex”). To evaluate our model's performance, we held out each time point and sex at a time, and we used profiles from the opposite sex to recapitulate the held-out profile. Sunbear is able to predict scRNA-seq profiles in an unseen time point in the opposite sex, with significant improvement compared with using baseline models of the previous and/or subsequent time points in the held-out sex (Fig. 2D,E).

To test whether Sunbear can operate on data sets with sparser time points, we performed two additional experiments: (1) iteratively holding out the target time point's neighboring one, two, or three time points (Supplemental Fig. S3) and (2) making the data sets sparser with gaps of 0.5, 0.75, and 1 day (Supplemental Fig. S4). Our result shows that the method is robust to data with sparser time points.

Furthermore, we compared Sunbear with TrajectoryNet on the sparser data set with a 0.75 day time gap. This analysis showed that Sunbear also outperforms TrajectoryNet in inferring the held-out time point profile (Supplemental Fig. S5; Supplemental Methods, “Comparison with TrajectoryNet”).

We further used Sunbear to integrate three sets of single-cell RNA-seq time-series profiles (Cao et al. 2019; Pijuan-Sala et al. 2019; Qiu et al. 2022). To assess how well the integration worked, we quantified LISI scores (Korsunsky et al. 2019) between each pair of neighboring time points, as well as between data sets collected at matched time points. Our results suggest that Sunbear can accurately align single-cell time-series profiles across time points and batches (Supplemental Fig. S6).

Finally, we performed several ablation studies on different parts of the model. To assess the contribution of the sinusoidal encoding of time information, we compared Sunbear with a variant of Sunbear that encoded time factor as a single value representing the actual time point. We observed decreased temporal inference performance in the latter scenario (Supplemental Fig. S7A,C,E). Similarly, removing the condition factor decreases the performance of temporal inference (Supplemental Fig. S7B,D,F). When removing the batch factor from the model, we observed a misalignment of cell identity factors across different data sets, compared with well-aligned cell identity factors from the original Sunbear model (Supplemental Fig. S8). These results suggest the importance of explicitly encoding condition and batch factors and representing time with a sinusoidal encoding.

### Sunbear reveals sexual dimorphism in gene expression during mouse embryonic development

Having established Sunbear's ability to capture sex differences in gene expression, we used the model to investigate sex-specific transcriptional differences during mouse embryonic development. Because only one sex is profiled at most time points, it is impossible to directly perform differential expression analysis between sexes at individual time points. Therefore, for each cell, we swapped the sex factor and calculated differential gene expression between sexes (see the Methods subsection “Predicting transcriptional sex differences across time”).

**Figure 3.**
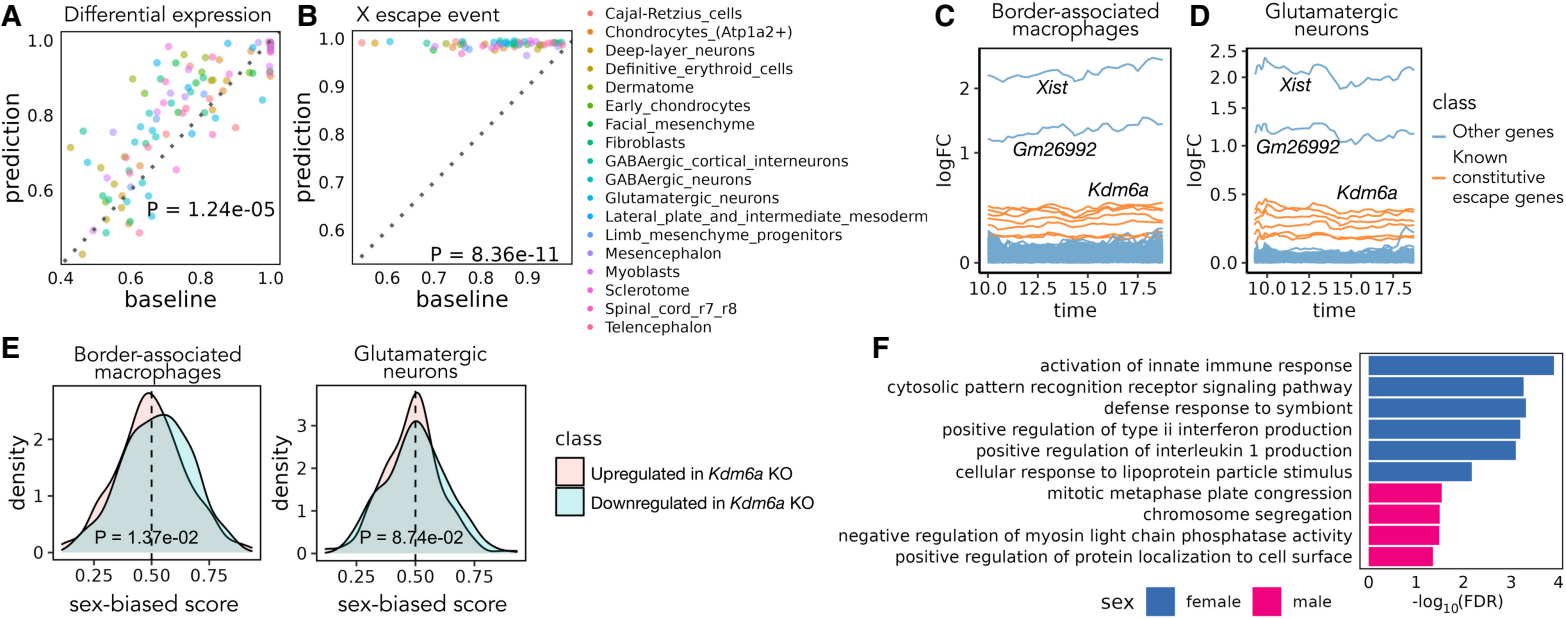
Sex differences in mouse embryonic development. (*A*) Pairwise comparison of Sunbear prediction and the nearest neighbor baseline in recapitulating differential expression patterns in sex-matched time points. Each dot indicates the AUROC score of recapitulating female-/male-biased patterns in each sex-matched time point and cell type. (*B*) Similar to in *A*, pairwise comparison of Sunbear prediction and the nearest neighbor baseline in ranking escape genes to be more female-biased than all other genes on the X Chromosome. (*C*,*D*) Predicted temporal sex-biased log fold change in glutamatergic neurons and border-associated macrophages. Each line represents a gene that is predicted to be consistently higher in females than males and is colored by whether the gene is a known constitutive escape gene or not. (*E*) Distribution of predicted sex-biased scores of genes (0 = extremely male-biased, 1 = extremely female-biased), grouped and colored by whether the gene is upregulated (pink) or downregulated (blue) in *Kdm6a* KO versus WT samples in CD4^+^ cells. *P*-values are calculated by one-sided Wilcoxon rank-sum tests. (*F*) Gene Ontology biological processes enriched in consistently female- and male-biased genes in border-associated macrophages. Nonredundant terms with the smallest FDR are selected for visualization. No enrichment of biological processes is found in glutamatergic neurons.

We performed two types of validation of the transcriptional sex-difference predictions, taking advantage of the three time points (E13, E13.25, E13.75) for which the original data include both female and male samples. Specifically, we performed evaluation on each cell type that has more than 1000 cells profiled across the three time points. First, we compared our prediction against the differential expression pattern between sexes calculated based on the original data at each sex-matched time point. For each of the three time points, we used the area under the receiver operating characteristic curve (AUROC) to assess the extent to which Sunbear's sex-difference prediction identifies the female- and male-biased genes derived from the original data set. As a baseline, we used the differential expression pattern from the closest time point as the sex-difference prediction for the time point of interest (see the Methods subsection “Validation of sex-difference prediction”). Comparing the two, we found that Sunbear significantly outperforms the baseline method (one-sided Wilcoxon paired rank-sum tests *P* = 1.24 × 10^−5^) (Fig. 3A). In the second validation, we compared both the predicted and original sex difference patterns against a small set of genes known to escape X Chromosome inactivation (XCI) in most/all mouse tissues (known constitutive escape genes; see the Methods subsection “Validation of sex-difference prediction”) (Berletch et al. 2015). Escape genes are transcribed from both copies of the X Chromosome in females (XX) but only from the one X copy in males (XY); thus, they are expected to have higher expression in females than males. Sunbear is able to rank the known escape genes to be more highly female-biased than other genes on the X Chromosome (Fig. 3B). In contrast, these genes are not ranked highly when we perform differential expression analysis directly on the original data (one-sided Wilcoxon paired rank-sum tests *P* = 8.36 × 10^−11^) (Fig. 3B). This trend is consistent when comparing based on area under the precision-recall curve (Supplemental Fig. S9). These results suggest that Sunbear is able to capture time-specific and sex-biased transcription patterns.

We then systematically investigated the sex-biased pattern of two major cell trajectories across all time points: macrophages and central nervous system (CNS) neurons (see the Methods subsection “Validation of sex-difference prediction”). Specifically, we focused on the most abundant cell types within each trajectory: glutamatergic neurons and border-associated macrophages. Macrophages have been widely studied in the adult stage and exhibit well-known sex-biased transcription and phenotypes (Gal-Oz et al. 2019). On the other hand, sex differences in CNS neurons could be limited because the brain is the somatic tissue showing the least sexual dimorphism in gene expression (Yang et al. 2006; Oliva et al. 2020; Rodríguez-Montes et al. 2023). To our knowledge, no previous study has looked into sex differences in the expression of these cell types in vivo during embryonic development. Ranking Sunbear's prediction based on the median log fold change between females and males across all time points, the top female-biased gene is consistently *Xist*, which is a gene expressed exclusively from the inactive X Chromosome and is essential for the onset of XCI in females (Fig. 3C,D; Loda and Heard 2019). The second most female-biased gene is *Gm26992*, an antisense to *Xist* with an unknown function. This is followed by other constitutive escape genes, including *Kdm6a*, *Jpx*, *Eif2s3x*, *Pbdc1*, *Kdm5c*, *Ftx*, and *Ddx3x*. In addition to the eight to nine constitutive escape genes found in both cell types, macrophages show an additional 14 X-linked genes (out of a total of 356 genes with a female bias), whereas neurons only show three X-linked genes (out of a total of 226 genes with female bias) (Supplemental Table S1). This is consistent with the incomplete silencing of the X Chromosome in macrophages (Syrett et al. 2019).

Beyond sex-linked genes, Sunbear also predicts autosomal genes with consistent biases toward females or males in each cell type across time, although the predicted sex differences associated with these genes are associated with relatively small fold changes in gene expression (Supplemental Table S1; Fig. 3C,D). Examples of autosomal genes with a female bias in macrophages include *Fcgr2b*, *Samd9l*, and *Ccr5*, which have been implicated in autoimmune diseases (Verbeek et al. 2019; Robinson et al. 2022; Zhou et al. 2023). Fewer autosomal genes have consistent female bias in glutamatergic neurons, which show a few female-biased genes in common with macrophages (e.g., *Trim30a*, *Vmn2r29*). These genes show sex-biased transcription in embryos before sex hormones are produced, suggesting that they are regulated by sex-linked regulators within each cell.

We thus hypothesized that predicted female-biased autosomal genes are functionally associated with female-biased X-linked genes. To test this hypothesis, we focused on *Kdm6a*, which encodes a histone demethylase that removes the repressive histone modification H3K27me3 to activate gene expression and is predicted by Sunbear to be the escape gene with the largest female-biased expression among expressed escape genes (except for *Xist*) in multiple cell types. Specifically, we hypothesized that autosomal genes reported to be downregulated in a *Kdm6a* conditional knockout (KO) experiment in CD4^+^ cells (i.e., genes potentially activated by KDM6A) (Itoh et al. 2019) would also show female-biased expression in border-associated macrophages compared with those upregulated in *Kdm6a* KO (i.e., genes potentially repressed by KDM6A). Indeed, Sunbear identifies the expected trend in border-associated macrophages (one-sided Wilcoxon rank-sum test *P* = 1.37 × 10^−2^) (Fig. 3E). Meanwhile, we do not observe significant difference in neurons (*P* = 8.74 × 10^−2^), possibly owing to low or no expression of these genes in neurons or to different causes of sex bias in the two cell types.

Furthermore, we quantified the odds ratio of protein–protein interaction (PPI) between the consistently female-biased X-linked genes and autosomal genes using the STRING database (see the Methods subsection “Sex-biased gene and pathway analysis”) (Mering et al. 2003) and observed a significantly larger number of PPIs between female-biased autosomal genes and X-linked genes in macrophages compared with the number of PPIs between random sets of autosomal genes and female-biased X-linked genes (permutation test *P* = 9.90 × 10^−3^; glutamatergic neurons are not included in this analysis because there are very few PPIs between sex-biased genes). These findings indicate that sex-biased transcriptional changes are not limited to sex-linked genes and that autosomal sex-biased gene transcription may be influenced by sex-biased transcription of sex-linked genes.

Although predicted consistent female- and male-biased genes in glutamatergic neurons are not enriched for specific biological processes, female-biased genes predicted in border-associated macrophages show significant enrichment in immune-related processes (hypergeometric test with FDR ≤ 0.05) (Supplemental Table S1; for selected GO terms, see Fig. 3F). These findings agree with previous evidence suggesting that immune processes tend to be stronger in females (Klein and Flanagan 2016).

### Cross-modality temporal profile inference reveals dynamic chromatin priming patterns

Sunbear can also be applied to infer multimodal temporal profile changes. To do so, we trained Sunbear on scATAC-seq and scRNA-seq time-series profiles collected from overlapping time windows, including scRNA-seq (collected from E6.5–E8.5 with 6 h intervals) (Pijuan-Sala et al. 2019) and scRNA-seq and scATAC-seq coassays collected from four time points (i.e., E7.5, E8, E8.5, E8.75) (Argelaguet et al. 2022). In total, there are four time points for scATAC-seq and 10 time points for scRNA-seq. We did not include the Qiu et al. (2024) data set in this analysis because the authors staged embryos by somite count rather than embryonic developmental time between E8 and E10. Because scATAC-seq measurements are, in general, sparser and more expensive to generate than scRNA-seq measurements, we configured Sunbear to use scRNA-seq time-series data as a reference and trained with a similar strategy to the single-modality temporal inference model. The training of scATAC-seq model is guided by the scRNA-seq reference by encouraging colocalization of cell embeddings between corresponding cells in scRNA-seq and scATAC-seq, as well as by copying the time-relevant neural network layer learned from scRNA-seq to scATAC-seq. By doing so, Sunbear can jointly model time-relevant changes across data modalities (Fig. 1).

**Figure 4.**
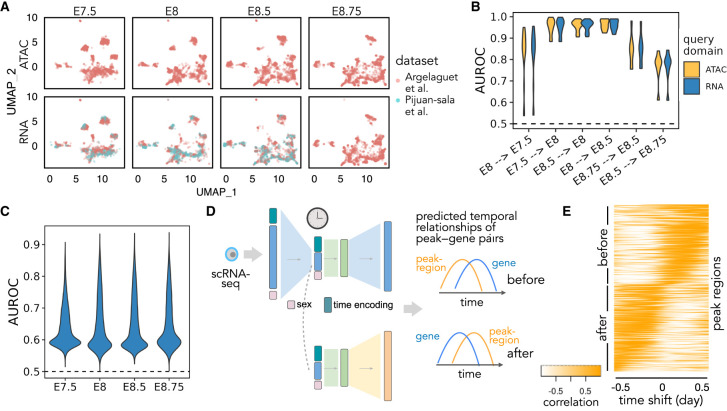
Multimodal temporal inference. (*A*) A UMAP embedding suggests that scRNA-seq and scATAC-seq profiles are well aligned across time and batch. Only time points with both scRNA-seq and scATAC-seq available are shown. (*B*) AUROC of the predicted differential accessibility pattern relative to those derived from the original data sets. AUROC is calculated per cell type, and differential accessibility is calculated between each held-out time point and each query time point (shown as “query time point → held-out time point”). (*C*) Peak-wise AUROC of scATAC-seq profiles predicted based on scRNA-seq relative to the original scATAC-seq profile in each held-out time point. AUROCs are calculated across all cells. (*D*) Workflow for calculating the dynamic association between peaks and genes. A query cell's scRNA-seq profile is fed into Sunbear to predict temporal patterns of gene expression and chromatin accessibility. For each pair of chromatin region and its proximal gene, we calculate the correlation coefficient between them with incremental time shifts, which results in a TLCC vector (column). (*E*) Visualization of predicted peak region–gene relationships. Heatmap of TLCC matrices on randomly selected 5000 peak regions with accessibility changes ahead of (“before”) or subsequent to (“after”) nearby gene expression. Peak regions are sorted based on the time shift with the maximum TLCC.

We first asked whether Sunbear can recapitulate the temporal patterns observed in the undercharacterized data domain (i.e., scATAC-seq) at a missing time point. To do that, we held out all scATAC-seq profiles from each embryonic developmental, and we trained Sunbear on the remaining cells. As a sanity check, we first used UMAP to visualize the cell embeddings from different data sets, modalities, and time points (Fig. 4A, with E8 held-out), verifying that cells from different batches and data modalities are colocalized. Then, using scRNA-seq or scATAC-seq profiles in neighboring time points as queries (e.g., E7.5 or E8.5 for E8), we applied Sunbear to predict each cell's chromatin accessibility in the held-out time point. Validating our prediction on the differential accessibility pattern between the held-out and each neighboring query time point per cell type, we found that Sunbear can accurately predict the temporal direction of chromatin accessibility changes (see the Methods subsection “Multimodal temporal inference evaluation”) (Fig. 4B; Supplemental Fig. S10). Notably, temporal accessibility can be recapitulated even when we use scRNA-seq profiles as queries. These observations demonstrate Sunbear's ability to capture cross-modality temporal patterns in missing time points.

Next, we assessed Sunbear's ability to recapitulate cell-to-cell variations in the missing data modality (i.e., scATAC-seq) and time point. To do that, we used scRNA-seq profiles from the held-out time point as queries to predict each cell's corresponding chromatin accessibility profiles. We then compared the predicted profile against the original scATAC-seq measurements by comparing the predicted and true scATAC-seq profiles per peak region across all cells (Fig. 4C), and we found accurate prediction of accessibility trends in >99.9% of peaks across all time points (see the Methods subsection “Multimodal temporal inference evaluation”).

Having validated Sunbear's performance on multimodal temporal inference, we trained the model on all time points, with the aim of characterizing patterns of temporal coordination between transcription and chromatin accessibility changes within each cell. Here, we focused on the hindbrain trajectory around E8.25, when the cell lineage first emerges (Qiu et al. 2022). Because there are no scATAC-seq profiles available at E8.25, we used scRNA-seq profiles collected in E8.25 in the hindbrain trajectory as queries to predict temporal cellular transcription and accessibility profiles (Fig. 4D). We then used time-lagged cross-correlation (TLCC) analysis (Chatfield 2013) to quantify the dynamic coordination between the predicted gene transcription and its nearby region's accessibility changes (Fig. 4D). Sorting genomic regions based on their temporal association pattern with nearby genes, we found diverse dynamic coordination between gene expression and chromatin priming (see the Methods subsection “Identifying temporal dynamics between chromatin priming and transcription”) (Fig. 4D,E). In particular, we observe a continuous gradient of time lags between chromatin accessibilities and their nearby gene expression changes. These patterns can be categorized as peak regions changing before or after gene expression changes.

We further asked whether distinct sequence motifs are enriched in genomic regions where Sunbear predicts peak accessibilities change before and after transcription changes. This analysis showed that several transcription factor binding motifs are enriched in genome regions in the “before” category (e.g., CTCF, ZIC2, and ZIC3) (Supplemental Fig. S11) but showed none in the “after” category (for details, see the Methods subsection “Identifying temporal dynamics between chromatin priming and transcription”). Given that transcription initiation requires the opening of chromatin regions at the enhancer and promoter regions and the recruitment of transcription factors, our finding agrees with a model in which chromosomal regions tend to be primed with transcription factors before transcription changes (Bernstein et al. 2006; Rada-Iglesias et al. 2011; Lara-Astiaso et al. 2014). Among the transcription factors that we predicted to prime chromatin regions before transcriptional changes, ZIC2 and ZIC3 are important transcriptional regulators of hindbrain segmentation (Elms et al. 2003; Drummond et al. 2013). Meanwhile, CTCF is a well-known mediator for chromatin loops and has been suggested to indirectly affect the regulation of genes involved in hindbrain development (Franke et al. 2021). Overall, the above evidence suggests that Sunbear is able to identify temporal coordination between chromatin priming and transcription based on static snapshots of cells measured in one modality and that Sunbear can offer insights into transcription factors involved in lineage priming during development.

## Discussion

A critical question for characterizing single cells is to understand how each cell's profile changes across time in vivo. Although most methods focus on inferring cell trajectories across time points collected from a single modality or condition, questions remain about how to model continuous profiles, compare profiles across biological conditions with unmatched time points, and understand the coordination between multiomic features. Sunbear provides a deep learning framework for temporal profile inference across data modalities and conditions. The Sunbear model jointly integrates data sets collected from different time points, batches, conditions, and modalities, and the model outputs continuous cellular profile changes across time. Sunbear thus allows us to directly compare cellular profiles across conditions and investigate joint temporal relationships between modalities. Furthermore, Sunbear can be trained across millions of cells and the full space of measured features (e.g., genes and peaks).

There are several advantages of Sunbear compared with combining different models together for temporal prediction. First, regarding the canonical example (i.e., using cross-modality prediction first followed by the temporal analysis), a major technical concern is the difference in data distribution between the observed and predicted values. Because single-cell RNA-seq and ATAC-seq are count-based and have a lot of missing values, single-cell analysis methods (e.g., cross-modality prediction frameworks) have been developed to take in these sparse and noisy measurements and output a denoised and smoothed prediction. Thus, real observations and cross-modality predictions have major differences and likely cannot be directly combined for downstream temporal inference methods. Second, Sunbear is able to incorporate single-cell time-series from partially unmatched time points across conditions or modalities, and it simultaneously optimizes for temporal inference and cross-modality inference, in which a cell's profile is estimated with the constraint that it should fall on a temporal trajectory and agree with its observed profiles if measured in another condition or data modality.

Compared with temporal methods on bulk measurements, Sunbear can capture cell-to-cell variation (as demonstrated in Fig. 4C), and this facilitates subsequent prediction of temporal coordination of gene and peak at cellular resolution (Fig. 4D,E). In addition, one of the key advantages of Sunbear is that it does not require knowledge of cell-type correspondence between time points. This is particularly useful for scenarios without clear knowledge of cell identity and cell-type correspondence (e.g., during embryonic development). For bulk-level analysis, it is technically challenging to separate individual cell types during the sample collection stage (e.g., during embryonic development); if this approach is applied to single-cell data sets, then the pseudobulk profiles rely heavily on prior cell-type annotation and knowledge of cell-type matching between time points.

Studies of sex differences during embryonic development are few and often lack matched biological samples across time points. In addition, differential expression analysis at the cell-type level may suffer from biases owing to differences in sub-cell-type composition between conditions. Our method mitigates this bias by computationally inferring cellular profiles at missing time points and conditions and by performing cross-condition comparisons at single-cell resolution. Our predictions, for the first time, systematically characterized sex-biased expression patterns during mouse embryonic development. Besides recapitulating well-known sex-biased genes, we generated various data-driven hypotheses for downstream experimental validation in the future.

Sunbear also identifies dynamic associations between chromatin priming and transcription. Previous studies on multimodal measurements have found or assumed that chromatin priming tends to occur ahead of transcription changes (Ma et al. 2020; Wu et al. 2025). In our analysis, we instead focused on the scenario in which only one type of data modality is measured, and we computationally predicted the multimodal dynamic coordination in each cell. Our results not only agree with previous knowledge but also suggest diverse types of dynamic coordination (e.g., different time delays, as well as positive and negative correlations). With the increasing amount of effort being devoted to characterizing mouse embryonic development, we envision that our temporal predictions can be combined with genome annotations from corresponding cell types and time points in the future.

In the future, Sunbear could be improved in several ways. First, although our model does not fully rely on the assumption of consistent cell proliferation or death rate, incorporating an explicit model of cell death and cell proliferation could further improve the temporal alignment, as was done previously (Schiebinger et al. 2019; Yeo et al. 2021). Second, as a proof-of-concept analysis, our current multimodal model leverages some coassay data to guide the alignment of cells across modalities. Generalizing the framework to carry out cross-modality alignment without requiring coassay data would greatly enhance the applicability of Sunbear. In addition, compared with neuralODE-based methods, although Sunbear has the advantage of being able to train on larger numbers of cells and more accurately estimating cellular profiles, it requires multiple time points to guide the inference of temporal trends and assumes that changes between time points are relatively smooth. We anticipate that adding constraints learned from trajectory inference models and pseudotime information to Sunbear's time encoding framework will further improve its generalizability and performance.

In the future, we envision the Sunbear framework being applied to integrate and compare human disease conditions collected at varying time points, characterize sequential transcription factor activation, and reveal the dynamic multiomic coordination during cell fate specification.

## Methods

### Mouse embryonic development data

The single-cell RNA-seq data sets used in this paper are listed in Table 2. For each data set, we obtained the raw count-by-cell matrices. For the coassay data set from Argelaguet et al., only time points with at least two biological replicates in this data set were retained. For efficiency of model training on the Qiu et al. data set (Qiu et al. 2024), we randomly subsampled cells to retain 1 million cells. Because P0 samples exhibit a huge environmental shift relative to embryonic stages, the P0 samples are not included in this analysis. In addition, because embryos before E10 are staged by somite counts, we converted the somite number to developmental time points by assuming equal time intervals between somite stages (i.e., each somite stage has incremental time of 2(day)/34(somite)).

**Table 2.** Time-series data in mouse embryonic development

Assay	Strain	Sex	Start	End	Freq	Source
sci-RNA-seq	BL6	m&f	E8	E18.75	2–6 h	https://omg.gs.washington.edu/ (Qiu et al. 2024)
sci-RNA-seq	BL6	–	E6.5	E8.5	6 h	https://tome.gs.washington.edu/ (Pijuan-Sala et al. 2019)
sci-RNA-seq	BL6	m&f	E8.5	E9.5	somite	https://tome.gs.washington.edu/ (Qiu et al. 2022)
sci-RNA-seq	BL6	m&f	E9.5	E13.5	24 h	https://tome.gs.washington.edu/ (Cao et al. 2019)
Coassay: scRNA-seq & scATAC-seq	BL6	–	E7.5	8.75	∼6 h	https://www.ncbi.nlm.nih.gov/geo/ (GSE205117)(Argelaguet et al. 2022)

### Modeling scRNA-seq temporal profiles

The goal of Sunbear is to decompose observed cellular profiles into latent factors representing cell identity, batch or condition, and the time point when each cell is collected. In this way, we can predict a cell's profile across time and conditions by concatenating its cell identity factor with varying time and condition factors. Thus, Sunbear takes as input single-cell time-series profiles and reconstructs them by cell identity, time, and batch/condition factors. Specifically, to interpolate continuous shifts of single-cell profiles across time points that are not captured, Sunbear represents time with sinusoidal encodings; that is, the embedding for each time point *t* is a *d*-dimensional vector, represented as

$${\left[\begin{array}{ccc} \sin(w_{1}\cdot t),\cos(w_{1}\cdot t), & \ldots & ,\sin(w_{d/2}\cdot t),\cos(w_{d/2}\cdot t)) \end{array}\right]}^{T},$$

where *k* ∈ 0, 1, …, *d* − 1 and *w*_*k*_ = 2π/10000^2*k*/*d*^, and *t* is represented in the units of day.

The default dimensions for cell identity embedding and time encoding is 50. The dimension of the time-aware embeddings is the same as the cell identity embeddings. The dimension of hidden layers is determined as the geometric mean of the input dimension and the latent dimension.

Sunbear is optimized toward two goals: obtaining accurate reconstruction of the observed single-cell profiles and aligning cells collected across different time points by learning time-invariant cell identity embeddings. To achieve these two goals, Sunbear adapts the cVAE framework (Lopez et al. 2018), with conditions as sinusoidal-encoded time factor and one-hot encoded sample conditions (e.g., sex, batch). Sunbear assumes that scRNA-seq counts follow a zero-inflated negative binomial (ZINB) distribution and estimates the reconstruction loss as the log-likelihood of the ZINB distribution (Lopez et al. 2018). Given scRNA-seq from time points one to *T*, Sunbear minimizes the following loss function:

(1) $${\mathrm{loss}}_{\mathrm{RNA}}=-\mathrm{zinb}.\mathrm{loglik}({\mathrm{RNA}}_{\mathrm{true}},{\mathrm{RNA}}_{\mathrm{pred}})+D_{\mathrm{KL}}[Q(z|X,t)||P(z,t)],$$

where *z* represents the distribution of the learned cell identity embedding based on the input gene expression profile (i.e., *RNA*).

To further encourage cell embeddings to be independent of time, we also add a discriminator that tries to predict time points based on cell embeddings, and we perform an alternative training by iteratively minimizing the discriminator loss,

(2) $${\mathrm{loss}}_{\mathrm{DIS}}=\mathrm{CE}(\mathrm{discriminator}(z),t),$$

and the generator loss,

(3) $${\mathrm{loss}}_{\mathrm{GEN}}={\mathrm{loss}}_{\mathrm{RNA}}-{\mathrm{loss}}_{\mathrm{DIS}}.$$

During model training, we held out all cells from one time point as the test set. Validation and training sets were created by randomly splitting the cells in the remaining time points with a 1:4 ratio. When there were large numbers of cells or time points, to make sure the model does not overfit to a high abundance time point, we capped the number of cells in the validation set at each time point to be 2000. Once Sunbear is trained with the existing time points, we can estimate single-cell profiles for any missing time point by swapping the time vector to the corresponding time point. We can also swap the condition factor to predict a cell's profile if it were captured in another data condition (e.g., an opposite sex).

### Extension of Sunbear for multimodal temporal interference

Next, we extended Sunbear to jointly infer temporal profiles across data modalities based on single-modal and multimodal time-series profiles. Similar to the single-modality case, for each data modality, Sunbear learns cell embeddings that represent cell identities, which are conditionally independent of the time embeddings, conditions, and batch effects. For the scATAC-seq domain, we binarize the scATAC-seq profiles and assume that they follow a Bernoulli distribution, estimating the reconstruction loss using binary cross entropy (Ashuach et al. 2022). Thus, the scATAC-seq model is optimized with

(4) $${\mathrm{loss}}_{\mathrm{ATAC}}=-\mathrm{BCE}({\mathrm{ATAC}}_{\mathrm{true}},{\mathrm{ATAC}}_{\mathrm{pred}})+D_{\mathrm{KL}}[Q(z|X,t)||P(z,t)].$$

Because of the imbalanced time-series measurements across data modalities (e.g., scRNA-seq time-series usually cover more time points than scATAC-seq), we designed Sunbear to take one data modality with denser time points as a reference to guide the imputation of the data modality with sparser time points. To this end, we optimized Sunbear by using scRNA-seq as a reference and forced the scATAC-seq model to have a shared, time-relevant network layer and cell embeddings with the scRNA-seq model. By doing so, we enable the sharing of interaction effects among cells, time, and conditions across data modalities.

Sunbear does stepwise optimization during training. In the first step, we train the scRNA-seq time model with Equation 1, while fixing all weights in the scATAC-seq model. In the second step, we train the scATAC-seq time model with regard to the scRNA-seq reference (Equation 5), with weights in the time factors fixed to be the same as the scRNA-seq and with the scATAC-seq cell embeddings forced to fall close to the scRNA-seq cell embeddings. We leverage the one-to-one cell correspondence in coassay data sets to supervise the alignment of cell embeddings between data modalities. This is achieved with a mean-squared error (MSE) loss between corresponding cell embeddings, together with a translation loss between scRNA-seq and scATAC-seq coassay profiles:

(5) $${\mathrm{loss}}_{\mathrm{multi}}={\mathrm{loss}}_{\mathrm{ATAC}}+\lambda *\mathrm{MSE}({\mathrm{ATAC}}_{\mathrm{embedding}},{\mathrm{RNA}}_{\mathrm{embedding}})+{\mathrm{loss}}_{\mathrm{TRANS}}(\mathrm{ATAC}|\mathrm{RNA})+{\mathrm{loss}}_{\mathrm{TRANS}}(\mathrm{ATAC}|\mathrm{RNA}).$$

During training, Sunbear iteratively optimizes the scRNA-seq model and the scATAC-seq model, so that the scATAC-seq model can slowly adapt to the cell embeddings and shared interaction effects of the reference scRNA-seq model.

To validate the model, we held out all single cells from one time point as the test set, and we randomly split cells from the remaining time points into a validation set (1/5) and a training set (4/5). The number of cells in the validation set at each time point is capped to be 2000.

### Hyperparameter tuning

As with any deep learning model, Sunbear has a variety of hyperparameters that need to be tuned. Accordingly, we performed a grid search on the following hyperparameters:

- number of latent dimensions in the VAE ∈ {25, 50, 100},
- minimum wavelength of sinusoidal encoding ∈ {1}day, and
- MSE weight λ (when trained on multimodality data) ∈ {1, 100, 10000}.

The number of hidden layers was fixed at two, and the dimension of the time embeddings was fixed at 50. In mouse embryonic development studies, the minimum wavelength of the sinusoidal encoding is defined as 1 day, because certain cellular profiles may follow a circadian pattern. In general, this parameter should be tuned to scale according to the actual time point values or expected biological variations. For single-modality data, we trained one model for each of the three points in our hyperparameter grid. For multimodal model, we trained one model for each of the 3 × 3 = 9 points in our hyperparameter grid. We then selected the best-performing model based on the validation set. Ideally, the model should be able to reconstruct the observed profiles accurately using the neighboring time points, without relying on cell-type labels. Thus, we calculate the cross-time prediction's pseudobulk Pearson's correlation with the original profile, as well as the LISI score (Korsunsky et al. 2019) between neighboring time points, as a way to assess the ability of each model to generalize across time. In the single-modality model, we select hyperparameters based on the summed rank of cross-time pseudobulk Pearson's correlation, LISI score across all neighboring time points, as well as the LISI score between neighbors of the held-out time point. The summed ranks across metrics are further rescaled so that the best model has a rank of one. When multiple batches are available, we also include the LISI score between batches in the model selection criteria (Supplemental Fig. S12A). In the multimodality model, we select the best hyperparameters using the above criteria based on the nonreference (i.e., scATAC-seq) data modality, together with the cross-modality LISI score and cross-modality translation losses in both directions (Supplemental Fig. S12B).

### scRNA-seq temporal inference evaluation

We evaluate the model's ability to generalize to uncharacterized sex and time points through leave-one-out cross-validation; that is, for each time point and each sex, we hold out the entire cell population from training and predict its profile from neighboring time points (“cross-time”) or the opposite sex (“cross-sex”). Because of the disruptive nature of single-cell measurements, there is no ground truth for a single cell's profile at another time point. Therefore, for the purpose of validation, we instead leverage cell-type labels, as previously annotated by biological experts, and ask whether the pseudobulk gene expression of each cell type can be correctly recapitulated, compared with pseudobulk profiles from corresponding cell types in neighboring time points.

To evaluate at the cell-type level, for each cell type in the held-out time point, we predict its profile using the neighboring time point's profile in the same cell trajectory and compare the prediction accuracy using pseudobulk Pearson's correlation between the held-out observed value and the predicted value. Specifically, because our prediction corrects for the sequencing depth of single cells, to make a fair comparison, we calculate the pseudobulk profile by normalizing every single cell's sequencing depth first before averaging them across cells. Only cell types with at least 25 cells in the held-out time point are considered in this evaluation.

For each held-out sex and time point, we make predictions with four combinations of starting points: previous or subsequent time points in the corresponding (“cross-time”) or opposite sex (“cross-sex”). The performance in each of these settings is compared against a corresponding baseline, which is the pseudobulk Pearson's correlation of the held-out cell type with the cell-type-specific profiles in the corresponding previous or subsequent time points in the corresponding sex. We also compared Sunbear to a stricter baseline: the pseudobulk profiles of the corresponding cell trajectory in the previous and subsequent cell types. This is a difficult baseline to beat because the cell trajectory is annotated with regard to the evidence that the previous, held-out, and subsequent cells in the same cell type should follow a sequential trajectory. However, Sunbear has not seen cells in the held-out time point or any cell-type labels and is forced to make predictions only based on the previous or subsequent time point. Given that embryos collected before E10 are staged by somite number, for which clock time cannot be precisely determined, we only perform such evaluation for samples collected after E10. One-sided Wilcoxon paired rank-sum tests are conducted to compare the prediction performance with baselines.

### Predicting transcriptional sex differences across time

For each gene, Sunbear returns a sex-biased score based on sex-matched predicted profiles across cells within each cell type. To do that, first, we predict each cell's denoised profile in both sexes. Then, for each cell type, we calculate each gene's sex-biased pattern over time by performing, for each time point, a one-sided Wilcoxon signed-rank test between predicted gene expression in females and males across all cells. Along with each statistical test, a sex-biased score is calculated as the sum of positive ranks divided by the sum of all absolute ranks *n*(*n* + 1)/2. We rescale the score to the range [0, 1], with values close to 0.5 indicating minimal sex difference and scores of one and zero representing strongly female- or male-biased expression. To make sure the sex-biased is robust to outliers, we only calculated such a score when there are more than 50 cells within a cell type.

### Validation of sex-difference prediction

To validate Sunbear's sex-difference prediction, we first retrieved sex-biased transcriptional patterns from three time points (E13, E13.25, and E13.75) with matched female and male samples by Qiu et al. (2024). Specifically, 18 cell types with enough observations across these three time points (i.e., 1000 or more cells) are selected for downstream evaluation. Because there are no biological replicates available at each time point, following the suggestion of Squair et al. (2021), for each cell type, we calculated the original differential expression pattern between sexes by applying a Student's *t*-test on sequencing depth–corrected and log-normalized single-cell profiles. Genes expressed in <5% of cells are excluded from the differential expression calculation. Genes with Benjamini–Hochberg-corrected FDR ≤ 0.05 are labeled as significantly up- or downregulated genes.

We then validated Sunbear's sex-difference prediction against the original differential expression pattern within each time point and cell type. To mimic the scenario in which only one sex is profiled at a time point, we held out the male sample in the time point of interest from model training and then calculated sex-biased scores based on the predicted profiles. We tested female- and male-biased expression prediction separately. In each direction, genes that are significantly female-/male-biased (FDR ≤ 0.05) are assigned positive labels, and all other genes are labeled as negatives. AUROC is calculated for our prediction against differential expression labels. In the end, for each cell type and time point, two AUROC scores are calculated.

As a baseline, we calculated the AUROC of the original differential expression patterns in the closest sex-matched time point against the female-/male-biased gene labels in the time point of study. A one-sided Wilcoxon paired rank-sum test was conducted to compare the prediction performance between Sunbear and the baseline across all matched time points, cell types, and differential expression directions.

Besides comparing Sunbear's prediction with the original measurements, we also validated the predictions against prior knowledge of sex-biased transcription. To do so, we retrieved X-escape genes (including *Ddx3x*, *Kdm6a*, *Kdm5c*, *Eif2s3x*, *Pbdc1*, *Jpx*, *Ftx*, and *5530601H04Rik*) from Berletch et al. (2015) and calculated the AUROC of predicting these genes to be more female-biased than all other genes on the X Chromosome. For this analysis, we used the differential expression pattern between sexes in original measurements as a baseline. For each cell type and time point, AUROCs based on our prediction are compared against those based on baselines using a one-sided Wilcoxon paired rank-sum test.

### Sex-biased gene and pathway analysis

To systematically characterize sex differences across time and avoid biases introduced by individual samples, we used two additional strategies to ensure robustness. First, we trained 10 models with a random time point held out from training each time, and we took the median of sex-biased statistics across these models as the predicted sex-biased score. Second, instead of comparing sex differences based on one biological sample at each time point, we calculated sex differences across cells from the current, previous and subsequent time points. These approaches allow us to derive robust sex-biased calculations across multiple biological samples.

To identify sex-biased genes for each cell type, we focused on time points with more than 25 cells in that cell type. To focus on genes that are important in that cell type, for each time point in that cell type, we ranked genes by predicted mean expression level across sexes and retrieved the first half of highly expressed genes. Only genes that are consistently expressed across all time points in the corresponding cell type are included in downstream analysis. Sex-biased genes are determined as genes with sex-biased scores consistently falling above or below 0.5 across all time points. GO term enrichment analysis was performed using a hypergeometric test, and only those terms with Benjamini–Hochberg-corrected FDR ≤ 0.05 are reported.

PPI information from *Mus musculus* was downloaded from the STRING database (Mering et al. 2003). Because we are interested in the broad definition of interactions (i.e., including coexpression, shared pathways, etc.), we did not apply additional filtering to the score and type of interaction in the STRING database. The odds ratio was calculated as the ratio of the odds of interactions happening between female-biased autosomal and X-linked genes, compared with odds of female-biased X-linked genes interacting with non-female-biased autosomal genes. Statistical tests are only carried out when there are 50 or more PPIs. We then generated the null distribution by calculating the odds ratio of interactions between randomly sampled autosomal genes and female-biased X-linked genes. Random sampling is repeated 100 times, and the size of the random sample is equal to the number of female-biased autosomal genes.

### Multimodal temporal inference evaluation

We first tested whether Sunbear can recapitulate temporal changes in scATAC-seq at a missing time point. Because we do not have ground-truth data at the single-cell level, we instead asked whether Sunbear can correctly predict the direction of chromatin accessibility changes across time in each cell type. To do that, we held out all scATAC-seq profiles from one time point and used scRNA-seq or scATAC-seq profiles at a neighboring time point as queries to predict the missing time point's scATAC-seq profiles. For each cell type and each peak, we predicted peak accessibility changes between the missing time point and the query time point using the test statistic of the one-sided Wilcoxon paired rank-sum test (i.e., the sum of positive ranks divided by the sum of all absolute ranks). To make sure the prediction is robust to random initiation and data split during model training, we trained 10 models with different random seeds and took the median of differential peak accessibility statistics as the final prediction. We then compared our prediction against differentially accessible peaks between cells measured in the held-out and the query time point within the corresponding cell type. Only cell types with 25 or more cells in both time points were retained. Differential accessibility of the original profiles was calculated using edgeR on the pseudobulk level (Fang et al. 2021). Finally, we report the AUROC of our prediction against significantly up- and downregulated peaks (labeled as positives and negatives, FDR ≤ 0.05).

To validate Sunbear's performance at capturing individual-cell-level differences in the missing data modality, we used scRNA-seq profiles in the held-out time point as queries, and we asked how well their corresponding chromatin accessibility profiles can be recapitulated. For each genomic region that is accessible in >5% of cells in the held-out population, we calculate the AUROC of the predicted accessibility against the original binary accessibility pattern across all cells. Similarly, for each genomic region, we also calculate the *P*-value of its predicted accessibility to be higher in cells with peaks in the original measurement than those without, using a one-sided Wilcoxon rank-sum test. The fractions of peaks with Benjamini–Hochberg-corrected FDR ≤ 0.05 are reported.

Because AUPR emphasizes the correctness at the top of the ranked list, we also calculated AUPR on recapitulating differentially expressed peaks from the temporal prediction. To correct for the skew in AUPR measurement toward peaks with large positive proportions (PP, PP = #(cells with peak expressed)/#cells)). We then normalized AUPRC with AUPRnorm = (AUPR-PP)/(1 − PP), where zero represents the behavior or a random predictor, and one indicates a perfect predictor. In this work, we use AUROC and AUPRnorm as performance measurements.

### Identifying temporal dynamics between chromatin priming and transcription

To investigate the dynamic relationship between gene expression and chromatin accessibility, we varied the time factor for a query cell to predict continuous changes in transcription and chromatin accessibility. We chose to focus on the hindbrain trajectory because it first emerges at E8.25, when scATAC-seq profiles are not available. In addition, the hindbrain trajectory is conserved across vertebrates, thus enabling us to validate our results on findings derived from model organisms (e.g., zebrafish) (Gilland and Baker 1993). We also focused on genes with significantly increased or decreased expression levels in the hindbrain at E8.25 relative to its parent trajectory at E8. We reasoned that temporal regulation of these genes is likely critical for hindbrain formation (Qiu et al. 2022). For each gene, we retrieved its proximal peaks that are mapped to 200 kb flanking regions upstream of its transcription start site. Then we predicted the gene expression and proximal chromosome accessibility changes within a [7.5, 9]-day interval, and we calculated the TLCC of the gene with respect to each of its proximal peak regions.

The TLCC score is calculated via the following steps. For each peak region and gene pair, we shift the predicted accessibility profiles along the time axis at 0.01-day steps, considering shifts in the range [−0.5, 0.5]. Then, for each step, we calculate the Pearson's correlation between the predicted expression profile and the shifted temporal accessibility profile within the core [8, 8.5]-day interval. Thus, each peak region and gene pair in a cell can be represented as a 101-dimensional vector consisting of Pearson's correlations corresponding to the varying time shifts. Concatenating all pairs of genes and peak regions, we obtain a TLCC matrix with 101 by #region–gene pair dimensions.

To maintain single-cell resolution while avoiding potential noise generated from a single cell or a single model, we trained a set of models with 10 random seeds and took the median of the predicted gene expression values or peak accessibility values. For a query cell, we selected its four closest neighbor cells summarized across the 10 models. Specifically, for each model, we retain its top 25 nearest neighbors based on Euclidean distance on Sunbear's cell embeddings and then sum the ranks across 10 models to obtain the final ranking. Cells that are not among the top 25 neighbors in any of the runs are excluded from the nearest neighbor list. On top of the five cells, we used Sunbear to predict each cell's multimodal temporal TLCC matrices and concatenated the TLCC vectors across the five cells along the peak gene axis. Because not all proximal chromatin regions regulate transcription, we removed region–gene pairs with a maximum correlation of less than 0.5 and only retained peak region and gene pairs showing up in more than one cell after the filtering step. Finally, we categorize peak regions into two categories: those whose accessibility pattern consistently changes ahead of its nearby gene expression pattern (“before”) or subsequent to the gene expression pattern (“after”).

To identify sequence features specific for each category, we first retrieved all unique sequences from peak regions within each category and then used MEME-ChIP to generate enriched motifs for one set of genomics regions against the other (with “differential enrichment mode” mode) with default cutoffs (Machanick and Bailey 2011). Tomtom was used to identify transcription factors with *Q*-value ≤ 0.05 (Gupta et al. 2007).

### Software availability

The Apache-licensed Sunbear source code is available at GitHub (https://github.com/Noble-Lab/Sunbear) and as Supplemental Code.

## Supplemental Material

Supplement 1

Supplement 2
