# Supplementary material for: Multicondition and multimodal temporal profile inference during mouse embryonic development
Source: Genome Res. 2025 Oct;35(10):2339–51. doi: 10.1101/gr.279997.124 (PMC12487814; doi:10.1101/gr.279997.124)
Supplement: Supplement 1 [file Supplemental_Materials.zip › Supplemental/Supplemental_Fig_S1.pdf]

Supplementary figures

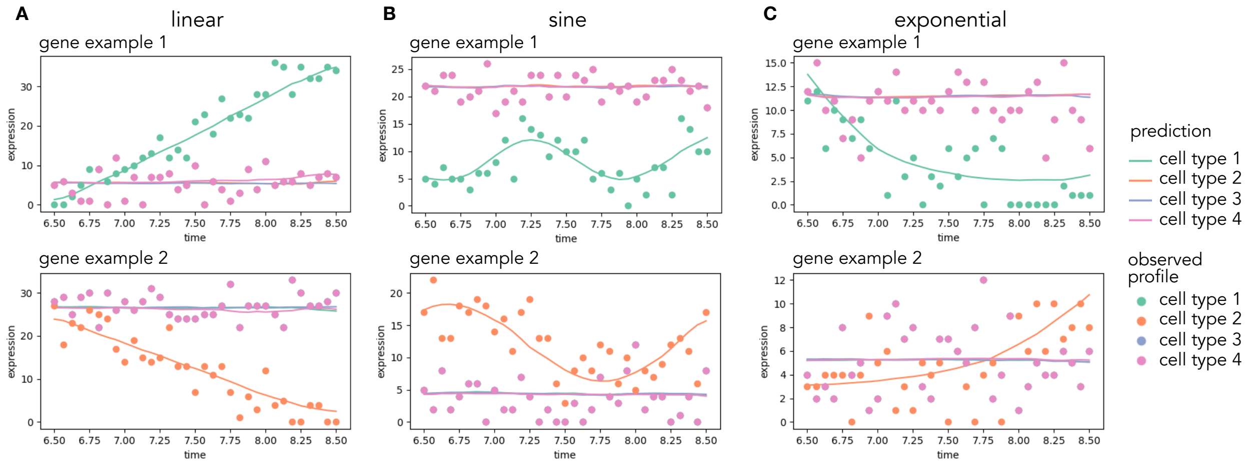

Supplementary Figure S1: **Prediction of temporal gene expression trajectory based on simulation.** (A) Observed (dot) and predicted (line) gene expression profile for the first cell in each of the four cell types. Gene expression is simulated to change either linearly or constantly with time. Top: an example of a gene changing linearly with time in cells from cell type 1, and constantly in other cell types. Bottom: another example of a gene that changes linearly with time in cells from cell type 2, and constantly in other cell types. (B) Similar to A, except the gene expression trend is simulated to be following a sine wave. (C) Similar to A, except the gene expression trend is simulated to be following an exponential trend.
