# Supplementary material for: Multicondition and multimodal temporal profile inference during mouse embryonic development
Source: Genome Res. 2025 Oct;35(10):2339–51. doi: 10.1101/gr.279997.124 (PMC12487814; doi:10.1101/gr.279997.124)
Supplement: Supplement 1 [file Supplemental_Materials.zip › Supplemental/Supplemental_Fig_S10.pdf]

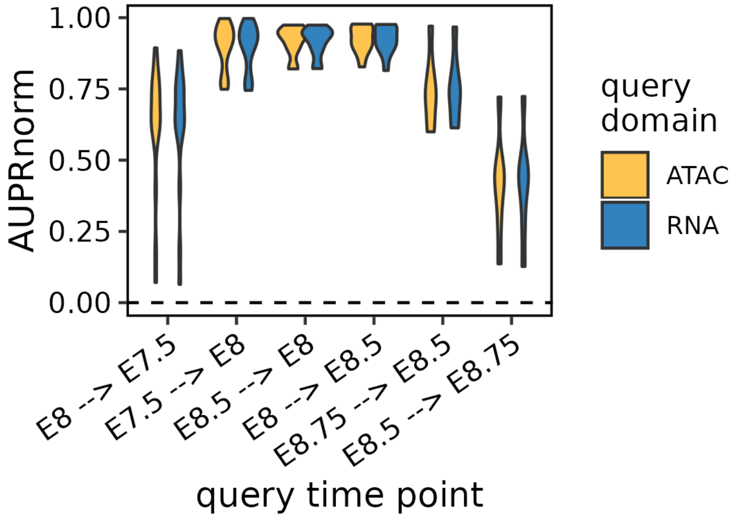

Supplementary Figure S10: **Evaluation of differential chromatin accessibility prediction across time** Normalized area under the precision-recall curve (AUPRC) of the predicted differential accessibility pattern relative to those derived from the original datasets. AUPRnorm is calculated per cell type, and differential accessibility is calculated between each held-out time point and each query time point (shown as "query time point → held-out time point").
