# Supplementary material for: Multicondition and multimodal temporal profile inference during mouse embryonic development
Source: Genome Res. 2025 Oct;35(10):2339–51. doi: 10.1101/gr.279997.124 (PMC12487814; doi:10.1101/gr.279997.124)
Supplement: Supplement 1 [file Supplemental_Materials.zip › Supplemental/Supplemental_Fig_S11.pdf]

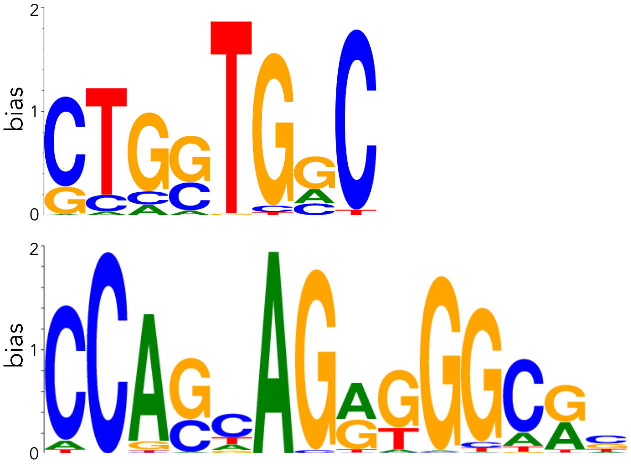

Supplementary Figure S11: **Enriched motifs for regions changing prior to gene expression trends.** Two motifs are enriched for regions changing prior to gene expression trends (E-value ≤ 0.05). Out of the two motifs, the upper one does not have significant transcription factor enrichment based on Tomtom (q-value $\leq$ 0.05). The lower motif has four transcription factors enriched: CTCF (q-value = 8.86e-08), CTCFL (q-value = 8.94e-06), ZIC2 (q-value = 4.29e-02), ZIC3 (q-value = 4.29e-02).
