# Supplementary material for: Multicondition and multimodal temporal profile inference during mouse embryonic development
Source: Genome Res. 2025 Oct;35(10):2339–51. doi: 10.1101/gr.279997.124 (PMC12487814; doi:10.1101/gr.279997.124)
Supplement: Supplement 1 [file Supplemental_Materials.zip › Supplemental/Supplemental_Fig_S12.pdf]

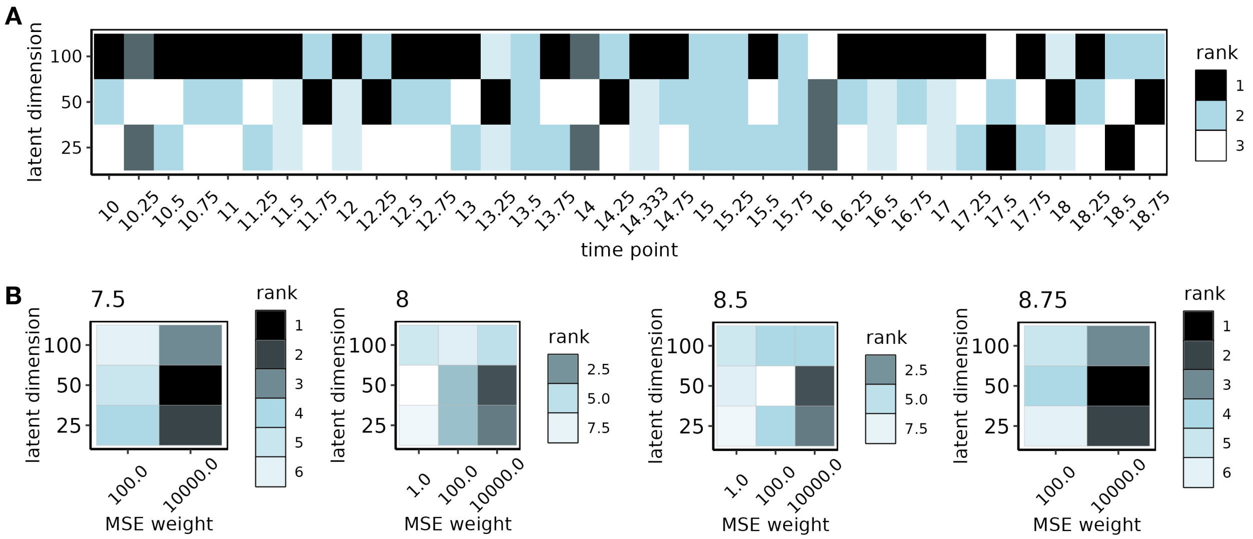

Supplementary Figure S12: **Hyperparameter selection based on the validation set.** (A) Rank of hyperparameters in each prediction task (x-axis), where one time point is held out and the rest of time points is used to train the scRNA-seq model. The rank represents the summarization of ranks of different evaluation metrics on the validation set (Section 4.4), where the model with the lowest rank is selected for downstream analysis. (B) Rank of hyperparameters in each prediction task (module) in the multi-omics setup. Within each module, the title represents the held-out time point, and rank based on the summarization of different evaluation metrics on the validation set is plotted across different choice of latent dimensions and MSE weights (Section 4.4).
