# Supplementary material for: Multicondition and multimodal temporal profile inference during mouse embryonic development
Source: Genome Res. 2025 Oct;35(10):2339–51. doi: 10.1101/gr.279997.124 (PMC12487814; doi:10.1101/gr.279997.124)
Supplement: Supplement 1 [file Supplemental_Materials.zip › Supplemental/Supplemental_Fig_S2.pdf]

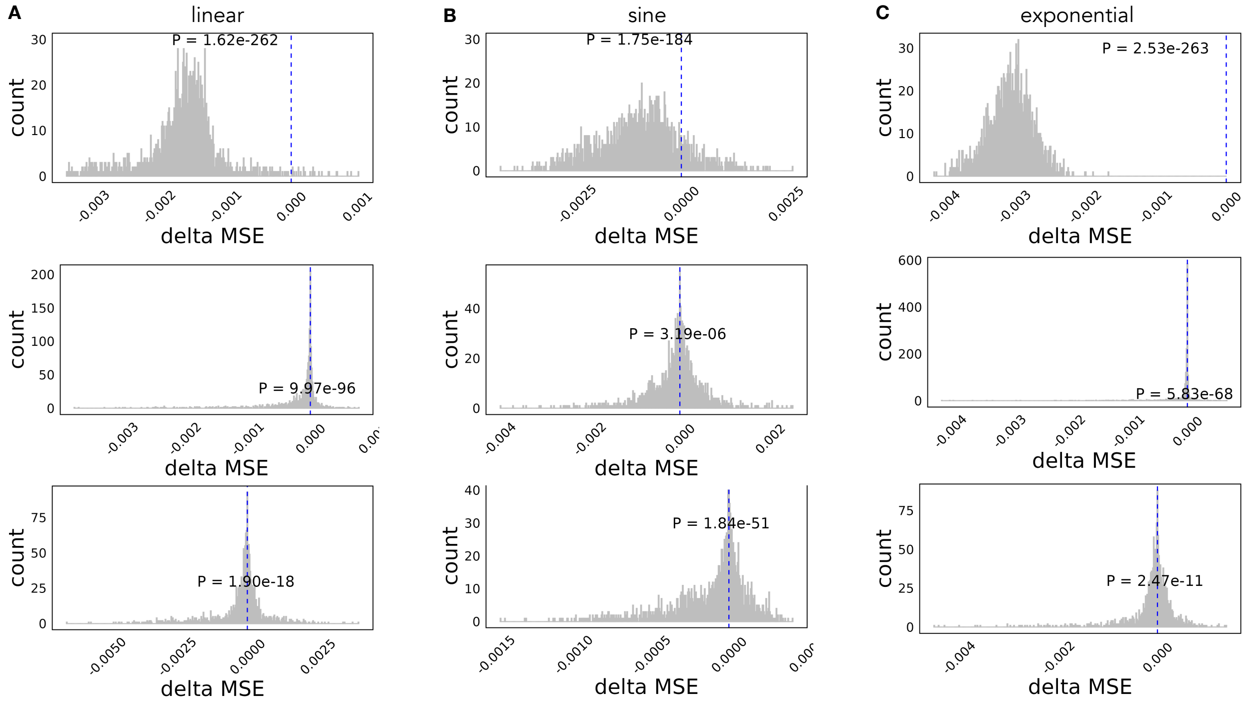

Supplementary Figure S2: **Evaluation of temporal prediction in cellular resolution based on simulation** (A) Difference of MSE for Sunbear prediction versus true value and MSE of the mean of neighboring time points versus true value. P-values are calculated by a one-sided Wilcoxon rank-sum test. (B) Difference of MSE for Sunbear prediction in the corresponding cell versus true value and MSE of Sunbear prediction in another cell from the same cell type versus true value. P-values are calculated by a one-sided Wilcoxon rank-sum test. (C) Difference of MSE for Sunbear prediction in the corresponding cell versus true value and MSE of Sunbear prediction in the corresponding cell versus true value of another cell from the same cell type. P-values are calculated by a one-sided Wilcoxon rank-sum test.
