# Supplementary material for: Multicondition and multimodal temporal profile inference during mouse embryonic development
Source: Genome Res. 2025 Oct;35(10):2339–51. doi: 10.1101/gr.279997.124 (PMC12487814; doi:10.1101/gr.279997.124)
Supplement: Supplement 1 [file Supplemental_Materials.zip › Supplemental/Supplemental_Fig_S3.pdf]

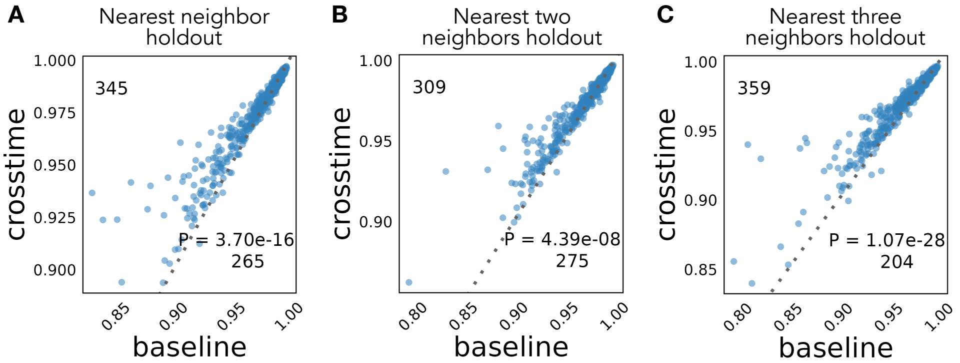

Supplementary Figure S3: **Sunbear outperforms baselines in cross-time evaluation when holding our more neighboring time points.** (A) Query and baseline are selected from the closest previous and subsequent time point out of the remaining time points. Pseudobulk Pearson correlation between the original held-out profile and predicted (y-axis) and baselines (x-axis) are plotted for each major cell trajectory in each held-out time point. Each dot represents a cell trajectory per held-out time point, and numbers indicate the number of dots above and below the diagonal line. P-values are calculated by a one-sided Wilcoxon rank-sum test. For each time point to be validated on, its the nearest (previous and subsequent) neighbors in the original dataset are held-out from training. (B) Similar to A, except the nearest two previous and subsequent time points are held out from training. (C) Similar to A, except the nearest three previous and subsequent time points are held out from training.
