# Supplementary material for: Multicondition and multimodal temporal profile inference during mouse embryonic development
Source: Genome Res. 2025 Oct;35(10):2339–51. doi: 10.1101/gr.279997.124 (PMC12487814; doi:10.1101/gr.279997.124)
Supplement: Supplement 1 [file Supplemental_Materials.zip › Supplemental/Supplemental_Fig_S4.pdf]

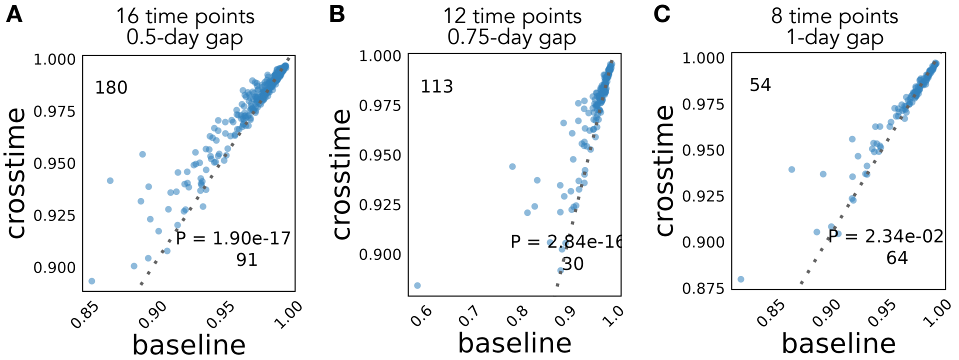

Supplementary Figure S4: **Sunbear outperforms baselines in cross-time evaluation on limited time points.** (A) Query and baseline are selected from the closest previous and subsequent time points. Pseudobulk Pearson correlation between the original held-out profile and predicted (y-axis) and baselines (x-axis) are plotted for each major cell trajectory in each held-out time point. Each dot represents a cell trajectory per held-out time point, and numbers indicate the number of dots above and below the diagonal line. P-values are calculated by a one-sided Wilcoxon rank-sum test. The original dataset is subsampled to have 0.5-day time intervals, i.e., 16 time points in total. (B) Similar to A, except the original dataset is subsampled to have 0.75-day time intervals, i.e., 12 time points in total. (C) Similar to A, except the original dataset is subsampled to have 1-day time intervals, i.e., 8 time points in total.
