# Supplementary material for: Multicondition and multimodal temporal profile inference during mouse embryonic development
Source: Genome Res. 2025 Oct;35(10):2339–51. doi: 10.1101/gr.279997.124 (PMC12487814; doi:10.1101/gr.279997.124)
Supplement: Supplement 1 [file Supplemental_Materials.zip › Supplemental/Supplemental_Fig_S5.pdf]

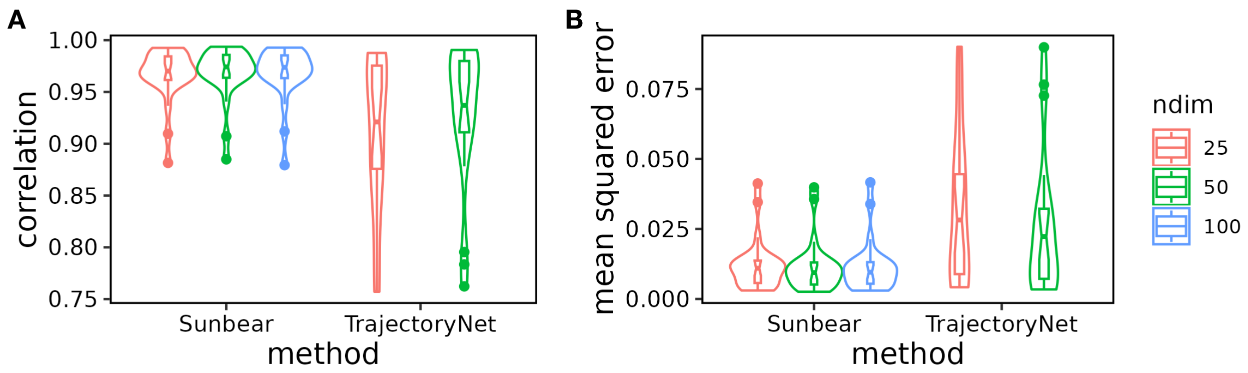

Supplementary Figure S5: **Performance comparison with TrajectoryNet.** (A) Violin plot of pseudobulk pearson correlation per major cell trajectory between the held-out profile (E17.5) and predicted profile using Sunbear or TrajectoryNet. All hyperparameters tuned are shown on the plot. (B) Similar to A, violin plot of mean squared error per major cell trajectory between the held-out profile (E17.5) and predicted profile using Sunbear or TrajectoryNet.
