# Supplementary material for: Multicondition and multimodal temporal profile inference during mouse embryonic development
Source: Genome Res. 2025 Oct;35(10):2339–51. doi: 10.1101/gr.279997.124 (PMC12487814; doi:10.1101/gr.279997.124)
Supplement: Supplement 1 [file Supplemental_Materials.zip › Supplemental/Supplemental_Fig_S6.pdf]

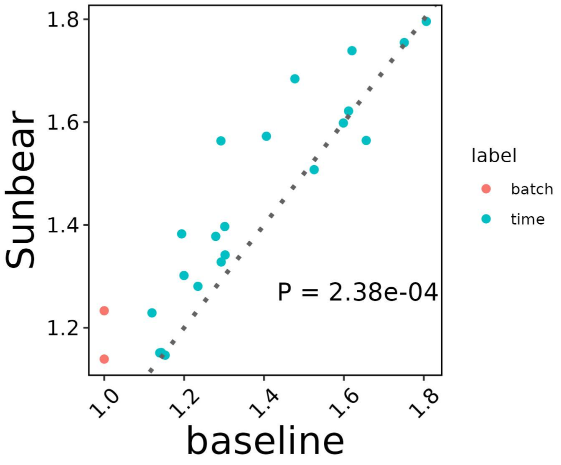

Supplementary Figure S6: **Alignment of multiple datasets across time.** LISI scores are calculated between each pair of neighboring time points (label: time) or between datasets on shared time points (E8.5 and E9.5, label: batch). LISI scores are calculated based on Sunbear's cell embeddings or baseline PCA embeddings of the original gene expression profiles. P-values are calculated by a one-sided Wilcoxon rank-sum test.
