# Supplementary material for: Multicondition and multimodal temporal profile inference during mouse embryonic development
Source: Genome Res. 2025 Oct;35(10):2339–51. doi: 10.1101/gr.279997.124 (PMC12487814; doi:10.1101/gr.279997.124)
Supplement: Supplement 1 [file Supplemental_Materials.zip › Supplemental/Supplemental_Fig_S7.pdf]

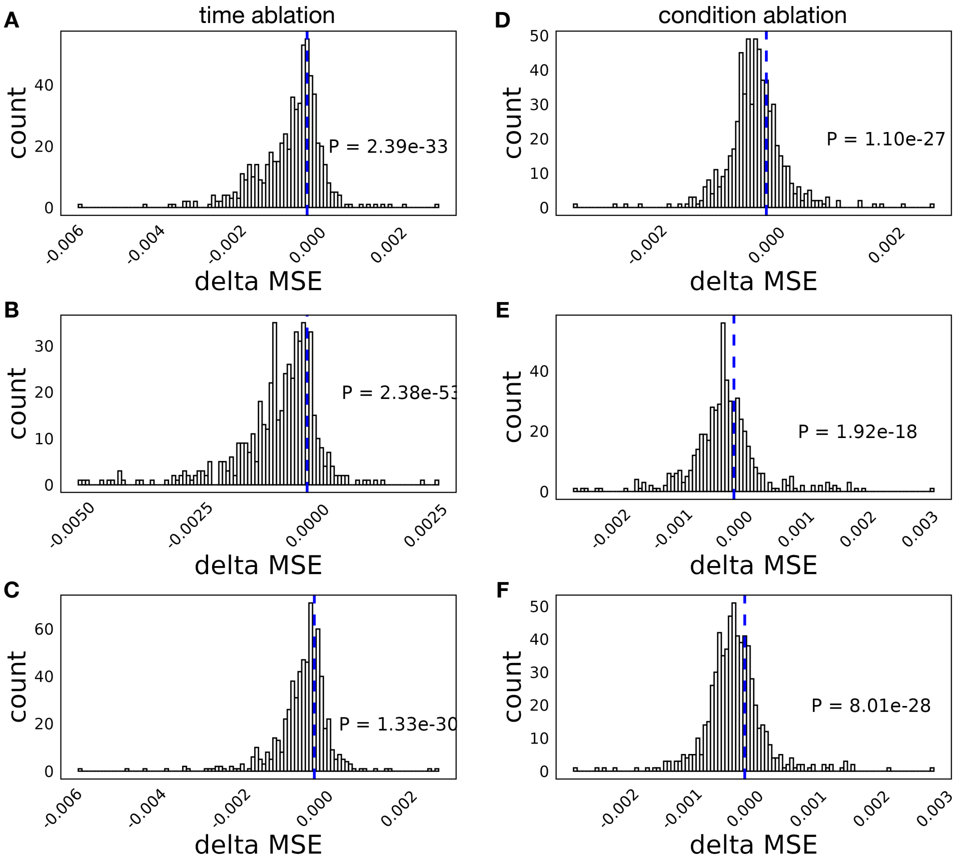

Supplementary Figure S7: **Performance comparison with time and condition factor ablated.** (A) Difference of MSE for Sunbear prediction versus true value and MSE of time-ablated Sunbear prediction versus true value. P-values are calculated by a one-sided Wilcoxon rank-sum test. (B) Difference of MSE for Sunbear prediction versus true value and MSE of condition-ablated Sunbear prediction versus true value. P-values are calculated by a one-sided Wilcoxon rank-sum test.
