# Supplementary material for: Multicondition and multimodal temporal profile inference during mouse embryonic development
Source: Genome Res. 2025 Oct;35(10):2339–51. doi: 10.1101/gr.279997.124 (PMC12487814; doi:10.1101/gr.279997.124)
Supplement: Supplement 1 [file Supplemental_Materials.zip › Supplemental/Supplemental_Fig_S8.pdf]

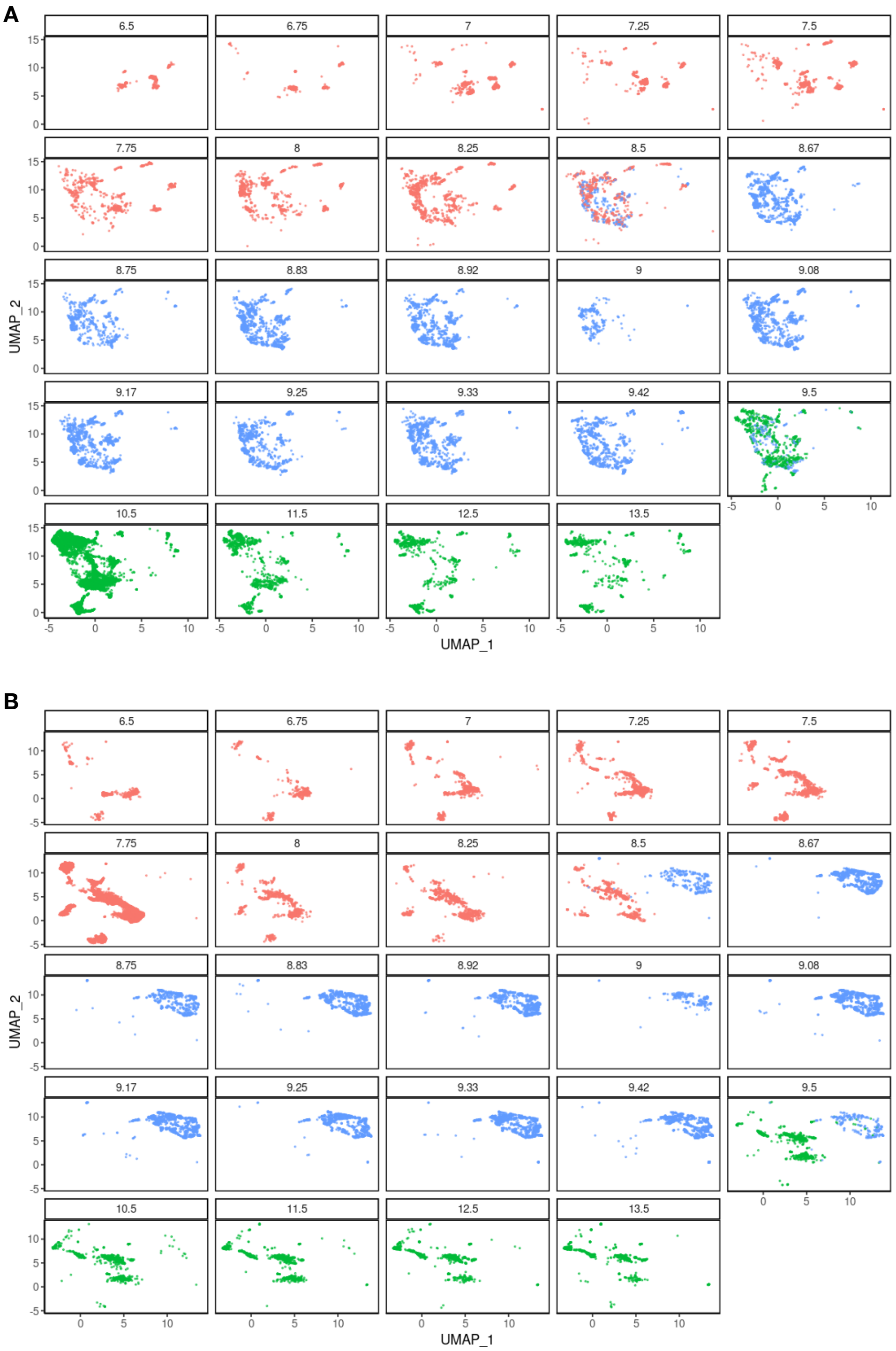

Supplementary Figure S8: **Cell identity embedding alignment with batch-aware and batch-agnostic model.** (A) UMAP of cell identity factors learned by Sunbear from three time-series datasets. Cells are colored by the dataset it is generated from. (B) Similar to A, except cell identity factors are learned by a variant of Sunbear that does not encode batch information.
