# Supplementary material for: Multicondition and multimodal temporal profile inference during mouse embryonic development
Source: Genome Res. 2025 Oct;35(10):2339–51. doi: 10.1101/gr.279997.124 (PMC12487814; doi:10.1101/gr.279997.124)
Supplement: Supplement 1 [file Supplemental_Materials.zip › Supplemental/Supplemental_Fig_S9.pdf]

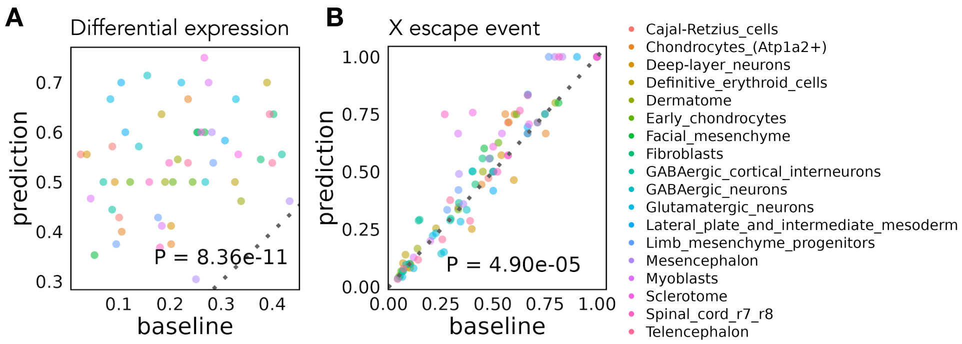

Supplementary Figure S9: **AUPRC on retrieving sex different genes.** (A) Pairwise comparison of Sunbear prediction and the nearest neighbor baseline in recapitulating differential expression patterns in sex-matched time points. Each dot indicates the area under the precision-recall curve (AUPRC) score of recapitulating female/male-biased patterns in each sex-matched time point and cell type. P-values are calculated by a one-sided Wilcoxon rank-sum test. (B) Similar to A, pairwise comparison of Sunbear prediction and the nearest neighbor baseline in ranking escape genes to be more female-biased than all other genes on the X chromosome.
