# Supplementary material for: Multicondition and multimodal temporal profile inference during mouse embryonic development
Source: Genome Res. 2025 Oct;35(10):2339–51. doi: 10.1101/gr.279997.124 (PMC12487814; doi:10.1101/gr.279997.124)
Supplement: Supplement 1 [file Supplemental_Materials.zip › Supplemental/Supplemental_Methods.pdf]

# Supplementary information

# Supplementary methods

## Simulation studies

We generated three simulation datasets representing three temporal patterns (linear, sine, exponential). The simulation proceeds in four steps.

1. Each dataset contains 2000 genes, 400 cells, and 33 time points (ranging from day 6.5 to 8.5, with a 0.0625-day time gap). Four cell types, each containing 100 cells, are simulated. Within each cell type, a unique set of 500 genes is assigned time-related profile changes, and the rest of the genes remain constant across time.

2. For each gene predefined to vary across time in a cell type, three parameters are randomly drawn from a distribution to define its temporal patterns. This step allows for gene-wise variation in temporal patterns:

   - $a \in \{-1, -0.9, \ldots, 1\}$
   - $b \in \{-1, -0.9, \ldots, 1\}$
   - $c \in [0, 30]$

   Depending on which type of temporal pattern is being modeled, the gene's expression is computed as follows:

   - constant: $x = |c|$
   - linear: $x = |20 * a * (t - 7.5 + sign(a))|$
   - sine: $x = |20 * b * (sin(5 * a * t + b) + 2)|$
   - exponential:
   $$x = 5 * b * \exp(2 * a * (t - 7.5)) \tag{S6}$$
   if x<0:
   $$x = x + |5 * \text{abs}(b) * \exp(2 * \text{abs}(a))| \tag{S7}$$

3. To allow for cell-level differences within each cell type, optionally, for each cell, we incorporated a time-shift factor $\delta$ to mimic the unsynchronized pseudotime of cells. The value of $\delta$ can be specified to account for different pseudotime spans. For the $i$th cell in a cell type, its pseudotime is then calculated as
   $$t_i = t - \delta * i \tag{S8}$$
   The cellular profile is then estimated based on the pseudotime of each cell, according to step 2.

4. We further added random noise from a Gaussian distribution to each expression value, and we rounded the expression value to a non-negative integer to mimic the noisy nature of scRNA-seq read counts:
   $$x = \text{round}(x + \epsilon) \tag{S9}$$
   where $\epsilon \in N(0, \sigma)$ and $\sigma$ is user specified.

We then held out one time point at a time, trained the model on the rest of the time points, and evaluated prediction performance on the held-out time point. Because we know the actual underlying gene expression value for each cell and time point, for each cell and gene we can evaluate the prediction by comparing the prediction with the actual value (without noise in step 3) using the mean squared error (MSE).

Three baselines were introduced to evaluate the single-cell level predictions of Sunbear:

1. We took the mean profile of the nearest previous and subsequent time points for the corresponding cell and computed MSE between the actual profile and mean baseline.

2. We randomly sampled a cell from cells in the same cell type and calculated the MSE of that cell's predicted profile with the actual profile of the original cell.

3. We calculated the MSE between the predicted profile and a randomly sampled cell's actual profile.

Because different genes and cell types can have dramatically different expression values, to avoid extreme values driving the evaluation, MSE is calculated based on log10(expression+1).

We also made several minor modifications to the model to accommodate this simulation study. In the real single-cell datasets, Sunbear's output is normalized by the sequencing depth of each cell, and the final gene expression prediction layer (the last layer in the decoder of the scRNA-seq model) uses a softmax function to enforce that. The simulation does not guarantee a dataset with comparable sequencing depth per cell. Thus, we opted not to perform sequencing depth correction in the model and swapped the activation function of the gene expression prediction layer from softmax to softplus. Similarly, to run on the simulation data with different scales and data distributions, we expanded the hyperparameter search to the following:

- learning rate $\in \{0.01, 0.001, 0.0001\}$
- number of latent dimensions in the VAE $\in \{4\}$
- minimum wavelength of sinusoidal encoding $\in \{1\}$
- discriminator weight across time $\in \{1, 100, 10000\}$

## Comparison with TrajectoryNet

TrajectoryNet is trained on a scRNA-seq dataset, where there is a 0.75-day interval between time points covering embryonic days 10 to 18.25. In total, there are 12 time points, 243,339 cells and 19,334 genes. Because TrajectoryNet traces cells from the last time point to previous time points, we held out E17.5 (the second-to-last time point), trained trajectoryNet on the rest of the time points, and used E18.25 (the last time point) to predict cellular profiles in E17.5. TrajectoryNet's interface allowed us to tune only the number of principal components to input to the TrajectoryNet model. We investigated values in $n \in \{25, 50, 100\}$ but found that $n = 100$ ran out of memory.

TrajectoryNet is trained and tested in the following steps. First, we hold out one time point as the test set and run PCA on sequencing-depth normalized and log-transformed data. Because we want to infer cellular profiles across all genes, PCA is performed across all genes. Then, we feed the top $n$ principal components to TrajectoryNet to train the model on the remaining time points. Next, to predict the held-out time point's profile, we use the last time point's latent representations to predict the previous time point's latent representations. We then project the inferred latent representation back to the original gene space by reversing the PCA transformation. Finally, we calculated pseudobulk gene expression profiles per cell trajectory in the missing time point, and we compare these predictions to the pseudobulk original profiles in the test set using Pearson correlation and MSE.

We trained Sunbear on the same dataset with the same hold-out and evaluation strategy.
